# IMMERSIVE GREEN SPACE AND EMOTIONAL BENEFITS FOR OLDER PEOPLE LIVING IN RESIDENTIAL CARE HOMES FOR ELDERLY (RCHES)

**DOI:** 10.1093/geroni/igae098.4312

**Published:** 2024-12-31

**Authors:** Hiu Kwan Yung, Adedayo Ogungbile, Eric Ohene, Yujun Fan, Siqiang Wang, Cynthia Yim

**Affiliations:** The Hong Kong Polytechnic University, Hong Kong, Hong Kong, China (People’s Republic); Hong Kong Metropolitan University, Hong Kong, Hong Kong, China (People’s Republic); The Hong Kong Polytechnic University, Hong Kong, Hong Kong, China (People’s Republic); The Hong Kong Polytechnic University, Hong Kong, Hong Kong, China (People’s Republic); The Hong Kong Polytechnic University, Hong Kong, Hong Kong, China (People’s Republic); The Hong Kong Polytechnic University, Hong Kong, Hong Kong, China (People’s Republic)

## Abstract

In densely populated cities like Hong Kong where land is scare, elderly residents in care homes often do not have access to natural gardens. However, previous research has proofed that natural gardens are beneficial to people’s psychological and social well-being. To address this problem, our study explores the use of a portable Cave Automatic Virtual Environments (CAVE) to simulate therapeutic effects of physical gardens, with the aim of providing elderly residents of RCHEs with the healing elements of nature, and potentially reducing stress and enhance emotional health. 18 residents living in two RCHEs were recruited to participate in the virtual garden experiments bi-weekly over two months. Questionnaire surveys were conducted to elicit their evaluation and feelings on the different features of the virtual gardens. We collected respondents’ skin conductance levels by using the Shimmer wearable sensors to analyze changes in heart rates, stress levels and emotional states. The results show that the virtual garden is able to make older people feel more relax and recall the nostalgic feelings of the built environment. This study has won the award organized by the National Academy of Medicine of the United States which recognizes its contribution in catalyzing innovations to promote people’s lifelong physical, mental, and social health and well-being.

